# Long-Term, Community-based Approach for Affected People Having Problems With Mental Health and Lifestyle Issues After the 2011 Fukushima Disaster: the Fukushima Health Management Survey

**DOI:** 10.2188/jea.JE20210178

**Published:** 2022-12-05

**Authors:** Masaharu Maeda, Mayumi Harigane, Naoko Horikoshi, Yui Takebayashi, Hideki Sato, Atsushi Takahashi, Maho Momoi, Saori Goto, Yuichi Oikawa, Rie Mizuki, Itaru Miura, Shuntaro Itagaki, Hirooki Yabe, Tetsuya Ohira, Seiji Yasumura, Hitoshi Ohto, Kenji Kamiya

**Affiliations:** 1 Department of Disaster Psychiatry, Fukushima Medical University School of Medicine, Fukushima, Japan; 2 Radiation Medical Science Center for the Fukushima Health Management Survey, Fukushima Medical University, Fukushima, Japan; 3 Department of Public Health, Fukushima Medical University School of Medicine, Fukushima, Japan; 4 Department of Gastroenterology, Fukushima Medical University School of Medicine, Fukushima, Japan; 5 Department of Neuropsychiatry, Fukushima Medical University School of Medicine, Fukushima, Japan; 6 Department of Epidemiology Fukushima Medical University School of Medicine, Fukushima, Japan; 7 Department of Blood Transfusion and Transplantation Immunology, Fukushima Medical University School of Medicine, Fukushima, Japan; 8 Research Institute for Radiation Biology and Medicine, Hiroshima University, Hiroshima, Japan

**Keywords:** Fukushima disaster, mental health, depression, posttraumatic stress disorder, problem drinking

## Abstract

A Mental Health and Lifestyle Survey (MHLS) has been conducted yearly as part of the Fukushima Health Management Survey since 2012, in order to monitor different health issues related to long-term evacuation of affected people after the 2011 Fukushima disaster. This survey is a mail-based one of nearly 210,000 affected people living in the evacuation zone at the time of the disaster. Another purpose of the MHLS is to provide efficient interventions by telephone based on the results of the survey. Significant findings contributing to understanding of non-radiological health effects caused by long-term evacuation were obtained from the MHLS, directly connecting to telephone-based interventions for over 3,000 respondents per year. In this article, the mental health outcomes of the MHLS, including depressive symptoms and posttraumatic responses, are reviewed, and the usefulness of telephone-based interventions is discussed. The evidence showed that, despite improvement of core mental health outcomes, the prevalence of respondents at high risk of some psychiatric problems remained high compared to that among the general population in Japan. In particular, several mental health consequences of respondents staying outside of Fukushima Prefecture were higher than those staying inside Fukushima. Along with further efforts to increase the response rate, we need to continue and modify the MHLS to meet the requirements of the affected people and communities.

## INTRODUCTION

The Great East Japan Earthquake, followed by the Fukushima Daiichi Power Station accident, forced numerous people living in Fukushima Prefecture to unexpectedly take up long-term evacuation life. In particular, the coastal area called “Hama-dori”, where the plant was located. was seriously affected, and, as a result, it was estimated that over 160,000 people were evacuated within the first year after the accident.^1^ Though the evacuation zone in Fukushima Prefecture had been quickly extended by governmental order, a huge number of people once living in the evacuation zone tried to relocate to as remote places as possible, even to Okinawa and Hokkaido in Japan. This was quite different from the evacuation pattern of those who were living in Miyagi and Iwate Prefectures who were mainly affected by the tsunami, where evacuees tended to stay behind within as close an area as possible from their original towns.^2^

Given past evidence regarding the mental health consequences of affected people after similar severe nuclear accidents, such as the Three Mile Island and Chernobyl accidents,^3^^,^^4^ there was an urgent need to establish a long-term support system to monitor and mitigate non-radiation-related health impacts, as well as radiation-related ones, such as thyroid cancer. In particular, mental health issues, including depression, posttraumatic stress disorder (PTSD), and suicide, were considered among the long-term consequences often seen after nuclear disasters.^3^^,^^4^ Amid the initial turmoil after the accident, mental health care plans had been examined by a few experts at Fukushima Medical University.

There were many difficult issues to be overcome at the time: for example, lack of health professionals, unclear prospects, budgets, and ethical considerations. The most difficult problem was that, whereas the number of local health professionals working in Fukushima had been insufficient even before the disaster, some left Fukushima due to worries about radiation-related health effects.^5^ In addition, another problem was the vastness of the target area and the population that needed to be taken charge of; that is to say, Fukushima Prefecture had a population of about 2,000,000 and the third largest area in Japan. Unlike natural disasters, boundaries between disaster and non-disaster areas were often vague,^2^ and, therefore, it was necessary to consider the view that all residents living in Fukushima Prefecture should be covered.

After much debate on measures that could be implemented for the affected people, it was eventually decided to focus on people living in the area designated as “the Evacuation Order Zone” by the Japanese Government, which had had about 210,000 original residents, most of whom were forced to evacuate, because they were more likely to have some mental and/or physical problems related to the unusual life under long-term evacuation. If the target area were broader (for example, including all residents of Fukushima Prefecture), it would not allow adequate care for the affected people due to the limited human resources available.

The actual survey and support that were launched as part of the Fukushima Health Management Survey was called the “Mental Health and Lifestyle Survey (MHLS)”.^6^^,^^7^ The MHLS was expected to contribute to monitoring and maintaining the comprehensive health status of affected people experiencing stressful lives after evacuation due to the disaster. Fully considering their negative feelings at that time about receiving “scientific examinations”, such as being “experimental animals”, it was emphasized that this survey would be closely connected with substantial support for the affected people and implemented as a “high-risk approach”. It was, therefore, required to be performed as an all-inclusive survey, not a randomly sampled one.

In the present paper, the survey protocol created through the above process and its results are shown in detail. The MHLS has many question items, and we have already published over 50 related articles in this last decade.^8^ In order to identify general trends in annual changes of different health outcomes among the target population, the focus was on several main outcomes, which were used to identify some mental health problems for the sake of the high-risk approach. The data are presented using a descriptive statistical method. In addition, the usefulness of the interventions provided for affected people based on the survey results is discussed. This paper should also help the readers understand that a severe nuclear disaster could have long-term and serious non-radiation-related effects on the health condition of the affected people.

## METHODS

### Survey design

#### Purpose

The purposes of the Mental Health and Lifestyle Survey (MHLS) were to identify the mental health and lifestyle-related issues of the target population and to provide them with appropriate support.

#### Target population

The target population of this survey was as follows:

1) Those registered as original residents in the areas designated as the Evacuation Order Zone as of April 1, 2012 after the disaster, even after moving out from these areas. This designated area included 13 municipalities: Hirono Town, Naraha Town, Tomioka Town, Kawauchi Village, Okuma Town, Futaba Town, Namie Town, Katsurao Village, Iitate Village, Minamisoma City, Tamura City, Kawamata Town, and parts of Date City specifically recommended for evacuation.
2) Those newly registered as residents in the above zones after April 1, 2012.

As noted above, the target population of the MHLS included people who relocated to the catchment area (the 13 municipalities) after the accident, as well as the original residents experiencing it. The reason was that, unlike natural disasters, the present accident could bring detrimental effects to people in the disaster area for a long period of time; thus, those relocating to this area after the accident might require professional support as well.

#### Procedure

Questionnaires developed for each age group were mailed to the target population yearly, with those for adults aged 16 years and above to be answered by themselves, and those for children aged 15 years and below to be answered by their parents/guardians; only those for adolescents aged 12 to 15 years were completed by both.

**Figure 1.**
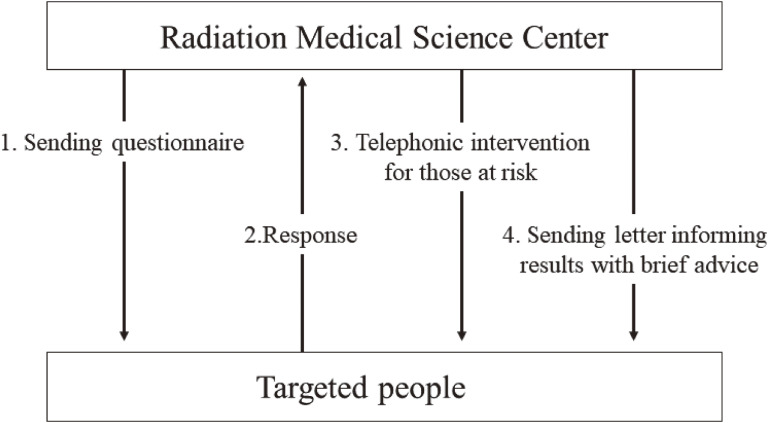
Procedure of the Mental Health and Lifestyle Survey (MHLS). The procedure of the MHLS is shown in the figure. The flow from sending questionnaires to providing interventions is in numerical order. The durations from 1 to 2 and from 2 to 3 are within about one month and within about 4 months, respectively. All procedures finish in about 10 months.

Forms explaining how the rights and privacy of participants were ensured were enclosed with the questionnaire. The replies were immediately checked. When respondents were identified as being at high risk of some mental health or lifestyle-related problems according to predetermined criteria, an attempt was made to contact them as soon as possible by telephone (if not possible, they were contacted by mail). For respondents who were not considered to be at high risk, letters informing them of their results with brief advice were sent on an individual basis (Figure 1).

The target population was divided into five groups by age group, as follows: 0–3 years, 4–6 years, 7–12 years (elementary school age), 13–15 years (junior high school age), and 16 years and above (adult age). Each was sent different questionnaires. The first survey was launched in January to February 2012.

#### Demographic and disaster-related variables

Data on sex, age, original address at the time of the disaster, current place of residence, current medical condition, medical history, damage to their house, evacuation situation, and other demographic and disaster-related variables were collected. As health-related variables, the participants were also asked about subjective health status, exercise habits, dietary pattern, sleep (satisfaction level, sleep duration), alcohol/tobacco consumption, and their other habits. The main measurements directly contributing to our interventions as described later are presented.

#### Main measurements

1) For adults (16 years and above)
  • Kessler’s six-item questionnaire (K6)^9^: To assess whether the respondent had depression or anxiety disorder, participants were asked six questions about how frequently the respondent felt depressed or anxious during the past 30 days. Those scoring 13 points or higher are considered to have potential depression or anxiety disorder in the Japanese version.^10^
  • PTSD Check List (PCL)^11^: To assess the severity of posttraumatic responses, participants were asked 17 questions on symptoms of posttraumatic stress disorder (PTSD) caused by the disaster during the past 30 days. This original Japanese version, standardized with MHLS data in 2017,^12^ was used during the first 3 years. It, however, was not used during the next 2 years to reduce the respondents’ burden. Thereafter, the PCL short version (4-item version: PCL-4) was newly standardized using the MHLS data,^12^ and it has been used since 2017. Cut-off points were 43/44 for the PCL and 11/12 for the PCL-4.^12^^,^^13^
  • CAGE (an acronym for attempts to Cut back on drinking, being Annoyed at criticisms about drinking, feeling Guilty about drinking, and using alcohol as an Eye opener)^14^: To assess whether the respondents had a drinking problem, participants were asked four questions about daily drinking behaviors during the past 30 days. Those scoring 2 points or higher were considered to have high-risk drinking.^14^ CAGE has been used only since 2013, not 2012.
  • Risk perception of radiation health effects: To assess risk perception of radiation health effects, participants were asked about the likelihoods of two types of radiation effects (delayed effects such as thyroid cancer or leukemia, and effects across generations such as genetic ones) using Lindell’s questions.^15^
2) For children (15 years and younger)
  • Strength and Difficulties Questionnaire (SDQ)^16^: To assess emotional and/or behavioral problems of children, parents/guardians who lived with participants were asked to complete 25 questions regarding such problems among their children during the past 6 months. Those scoring 16 points or higher in the Japanese sample were considered to require some psychological support or treatment.^17^

#### Ethical considerations

This study was approved by the Ethics Review Committee of Fukushima Medical University (Nos. 1316, 2148, 2795). It was explained in writing to the participants that their responses would not be published in any form that identified individuals. Participants who answered the self-administered questionnaires were considered to have consented to participate.

### Support system

#### Target population

As described above, a new support system that could provide efficient intervention for people at high risk of some health problems in the target population was established. Considering the expected number of high-risk people requiring professional support (estimated as at least 5,000 per year) and broad dispersal of the evacuees, interventions using the telephone were considered to be almost the only way to address them as quickly as possible. Thus, more than a dozen medical and welfare professionals, including public health nurses, clinical psychologists, and social workers, were gathered as “the Mental Health Support Team” and started to telephone respondents identified at high risk of some health problems based on the results of the MHLS, such as the K6, PCL, CAGE, and SDQ. Those having current lifestyle-related diseases, such as hypertension and/or diabetes mellitus, who did not receive any treatment were also targeted, regardless of necessity. Meanwhile, the criteria for the support have been changed slightly every year according to the situation. For example, higher cut-off points of the measurements, such as the K6, PCL, and SDQ, were adopted in the first survey year, because a great number of respondents were considered to need support at that time.

#### Intervention

The features of this brief telephonic intervention are not ‘on call,’ but rather ‘call service’ (Figure 1). In the context of a more positive, intensive approach to evacuees, it was called ‘phone support based on an outreach approach.’^18^ The Mental Health Support Team consisted of 15–17 counselors with more than 10 years of hands-on practical experience. These interventions for high-risk individuals included active listening, psychoeducation, and professional suggestions by telephone or mail. Each intervention usually took 10–30 minutes according to the conditions or demands of the target individuals, and it was mostly conducted between 9 AM and 5 PM on weekdays. Completing the intervention for all respondents took 6–8 months per year. Thus, the respondents who received this intervention were about 3,000–4,000 people yearly. The respondents were informed that they might receive a telephone call from the counselor if they were identified as being at high risk. This information was disclosed in a document that was part of the MHLS questionnaire.

#### Cooperation with other resources

If respondents needed more intensive care based on the telephonic intervention, the counselors referred them to adequate medical and/or welfare resources (eg, psychiatric clinic, local health center) after obtaining the respondent’s consent, as needed. To enrich such cooperation in communities, we have been trying to share the necessary information with key stakeholders, including prefectural and municipal governments. When a respondent was assessed to be in a crisis and need urgent help (eg, person having suicidal ideation) during telephone intervention, counselors strongly recommended visiting health experts and/or asked other care facilities to implement outreach services for him/her as needed.

## RESULTS

### Results of the survey

The results of the main measurements as described above among the many question items in the MHLS are primarily shown.

### Demographic data

In the first survey year (February 2012), a total of 210,189 people were mailed the survey, and the number of the target population decreased slightly to 203,827 in the 8^th^ survey (February 2019). Response rates obtained in the first year, 2012, were 40.7% for adults and 63.4% for children. Response rates, however, decreased yearly, to 19.9% for adults and 15.0% for children in 2019. The demographic data of the adults and the school-age children in 2012 and 2019 are shown in Table 1. The proportion of the adult respondents newly registered with the MHLS (those relocating to the catchment area after the accident) was 2.8% in 2019. In longitudinal studies regarding MHLS, they were basically excluded from the analyses except for the descriptive studies.

**Table 1.** Demographic and disaster-related variables in 2012 and 2019

	2012		2019	
**Elementary school students**				
Number of participants	7,464	(63.3%)	1,587	(16.0%)
Male	3,815	(51.1%)	796	(50.2%)
Female	3,649	(48.9%)	791	(49.8%)
Mean age, years	9.5		9.5	
Living place, present address				
Inside Fukushima Prefecture	5,404	(72.4%)	1,219	(76.8%)
Outside Fukushima Prefecture	2,060	(27.6%)	368	(23.2%)
Exercise frequency				
Almost everyday	932	(12.5%)	158	(10.0%)
Two to four times a week	1,495	(20.1%)	489	(30.9%)
About once a week	1,075	(14.4%)	421	(26.6%)
Almost never	3,950	(53.0%)	514	(32.5%)
Mental health problems				
SDQ ≥16	1,637	(22.0%)	156	(9.8%)

**Junior high school students**				
Number of participants	3,411	(56.1%)	756	(13.8%)
Male	1,717	(50.3%)	383	(50.7%)
Female	1,694	(49.7%)	373	(49.3%)
Mean age, years	14.0		13.9	
Living place, present address				
Inside Fukushima Prefecture	2,734	(80.2%)	602	(79.6%)
Outside Fukushima Prefecture	677	(19.8%)	154	(20.4%)
Exercise frequency				
Almost everyday	755	(30.2%)	209	(42.0%)
Two to four times a week	349	(14.0%)	93	(18.7%)
About once a week	221	(8.8%)	43	(8.6%)
Almost never	1,176	(47.0%)	153	(30.7%)
Mental health problems				
SDQ ≥16	539	(16.2%)	80	(10.8%)

**≥16 years**				
Number of participants	73,433	(40.7%)	35,905	(19.8%)
Male	32,301	(44.0%)	16,476	(45.9%)
Female	41,132	(56.0%)	19,429	(54.1%)
Mean age, years	55.5		63.2	
Living place, present address				
Inside Fukushima Prefecture	59,435	(80.9%)	31,035	(86.4%)
Outside Fukushima Prefecture	13,998	(19.1%)	4,870	(13.6%)
Sleep sufficiency				
Satisfied	17,587	(33.3%)	12,884	(41.1%)
Slightly dissatisfied	24,675	(46.8%)	14,333	(45.8%)
Quite dissatisfied	8,180	(15.5%)	3,432	(11.0%)
Very dissatisfied/could not sleep at all	2,312	(4.4%)	676	(2.2%)
Drinking status				
Never/rare drinker	37,286	(52.1%)	18,303	(54.2%)
Past drinker	2,720	(3.8%)	1,556	(4.6%)
Current drinker (more than once a month)	31,532	(44.1%)	13,881	(41.1%)
Psychological distress				
K6 ≥13	8,717	(14.6%)	1,756	(5.7%)
PTSD symptoms				
PCL ≥44	13,111	(21.6%)	N/A	
PCL-4 ≥12	N/A		2,651	(9.7%)

K6, Kessler 6-item Scale; PCL, Post-traumatic Stress Disorder Checklist; PTSD, post-traumatic stress disorder; SDQ, Strengths and Difficulties Questionnaire.

### General mental health conditions

**Figure 2.**
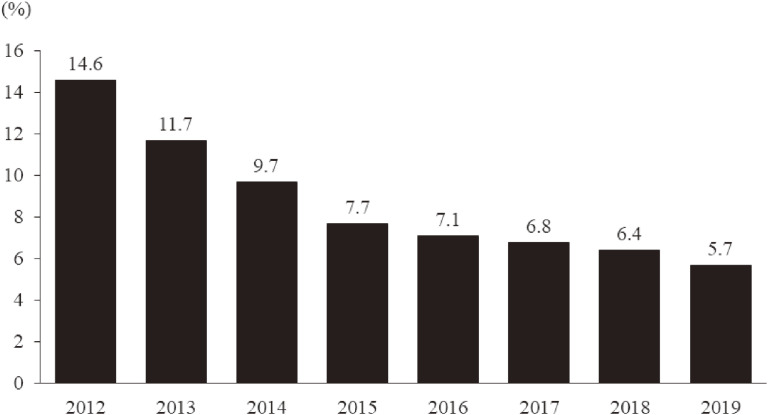
The prevalence of probable depression and anxiety disorder from 2012 to 2019. People with probable depression and/or anxiety disorder are identified using the Kessler 6-item Scale (K6) (≥13).

**Figure 3.**
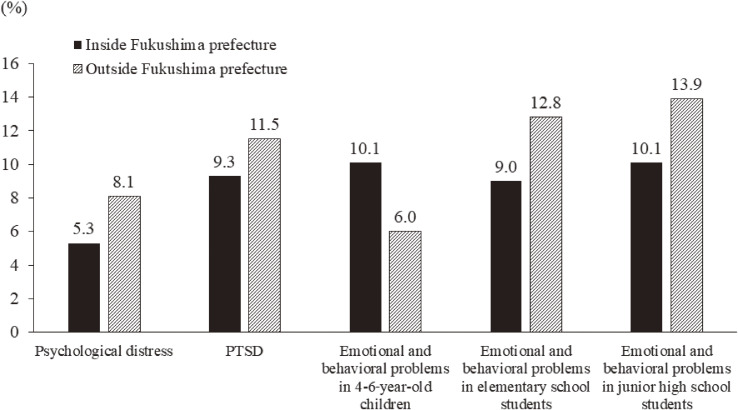
Comparisons between people living inside Fukushima and those living outside Fukushima in 2019. People with probable depression and/or anxiety disorder are identified using the Kessler 6-item Scale (K6) (≥13). The prevalence of probable PTSD is identified using the PCL short version (≥12). Children aged 4 to 15 years having some emotional and behavioral problems are identified using the Strength and Difficulties Questionnaire (SDQ) (≥16).

According to the K6 score, the prevalence of respondents having probable depression or anxiety disorder in 2012 was quite high (14.6%). However, it decreased yearly, especially during the first 4 years, reaching 5.7% in 2019 (Figure 2). In addition, this rate was higher among the respondents staying outside Fukushima than among those staying inside Fukushima in 2019 (8.1% vs 5.3%) (Figure 3).

### Posttraumatic responses

**Figure 4.**
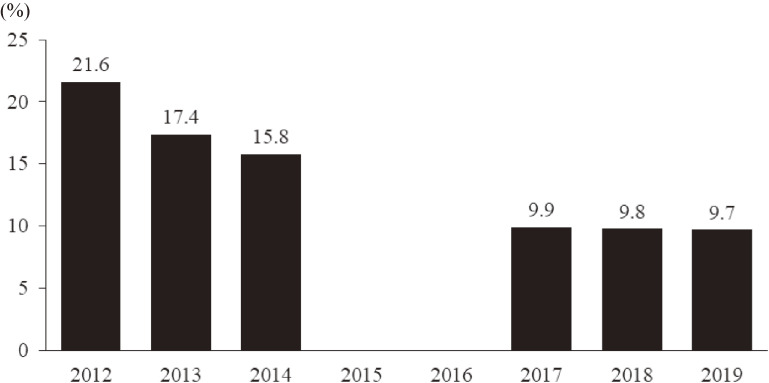
The prevalence of probable PTSD from 2012 to 2019. People with probable posttraumatic stress disorder are identified using the Post-traumatic Stress Disorder Checklist (PCL) from 2012 to 2014 (≥44) and the PCL short version from 2017 to 2019 (≥12). These measurements were not performed in 2015 and 2016.

The prevalence of respondents having probable PTSD was 21.6% in 2012, and it decreased during the next 2 years (Figure 4). After a 2-year interval, posttraumatic responses were assessed again using PCL-4, and the prevalence of those with probable PTSD was nearly 10% in the most recent 3 years (Figure 4). Similar to K6, the prevalence of those with probable PTSD was higher among the respondents staying outside Fukushima than among those staying inside Fukushima in 2019 (11.5% vs 9.3%) (Figure 3).

### Problem drinking

**Figure 5.**
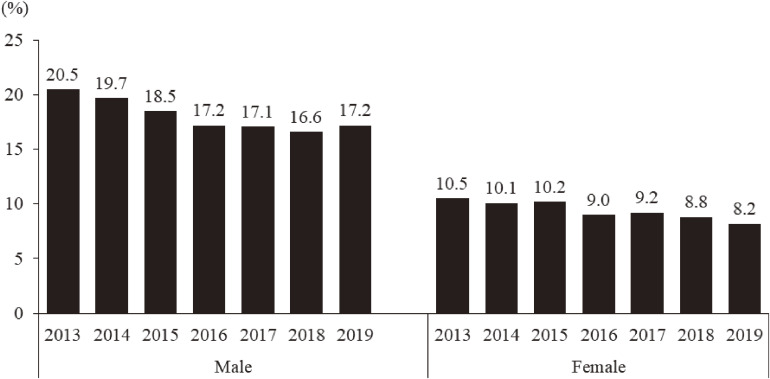
The prevalence of those at high risk of problem drinking from 2013 to 2019. People with a risk of problem drinking were identified using the Cutting down, Annoyed by criticism, Guilty feeling, and Eye-opener questionnaire (CAGE) (≥2).

Figure 5 shows the prevalence of respondents with problem drinking according to CAGE. The prevalence both in male and female participants decreased gradually from 20.5% in 2013 to 17.2% in 2019 in the male group, and from 10.5% in 2013 to 8.2% in 2019 in the female group (Figure 5). Unlike for the K6 and PCL-4, the prevalence of those with problem drinking was higher among the male respondents staying inside Fukushima than among those staying outside Fukushima in 2019 (17.5% vs 14.9%).

### Risk perception of radiation health effects

**Figure 6.**
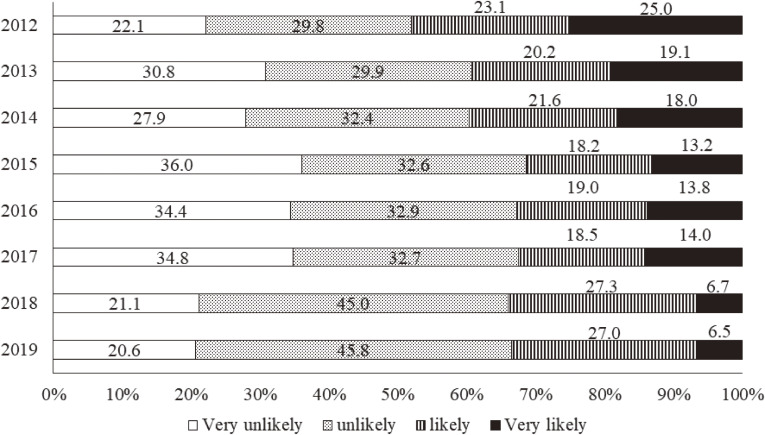
Radiation risk perception of delayed health effects from 2012 to 2019. The following question was asked: “Please describe your current understanding of how radiation exposure has affected your health. What do you think is the likelihood of damage to your health (eg, cancer onset) in later life as a result of your current level of radiation exposure?”

**Figure 7.**
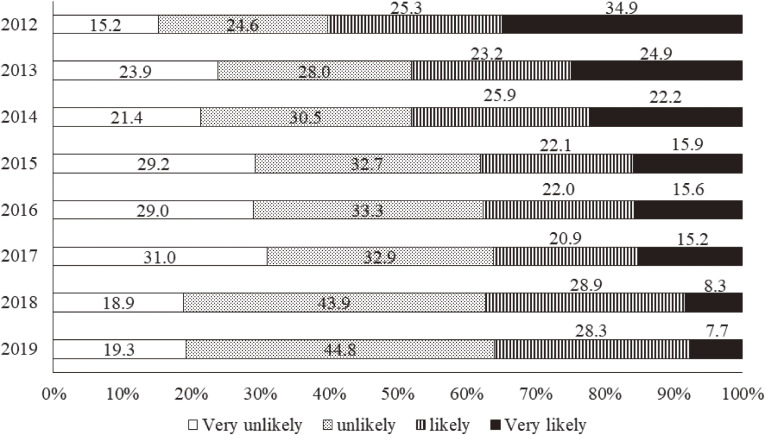
Radiation risk perception of health effects across generations from 2012 to 2019. The following question was asked: “Please describe your current understanding of how radiation exposure has affected your health. What do you think is the likelihood that the health of your future (ie, as yet unborn) children and grandchildren will be affected as a result of your current level of radiation exposure?”

According to Figure 6 showing respondents’ concerns about delayed health effects from the Fukushima disaster, 48.1% of the respondents answered that such health effects were likely or very likely to occur in 2012. Although this proportion decreased yearly during the first 4 years, it has remained almost unchanged in recent years, and nearly one-third still had concerns about such effects in 2019 (Figure 6). With regard to radiation health effects across generations, such as genetic ones, 60.2% answered that such health effects were likely or very likely to occur in 2012 (Figure 7). This proportion, similar to that for delayed health effects, decreased during the first 4 years, but remained unchanged in the recent 5 years as well. As a result, 36.0% still had concerns about such genetic effects in 2019 (Figure 7).

### Behavioral and emotional problems among children

**Figure 8.**
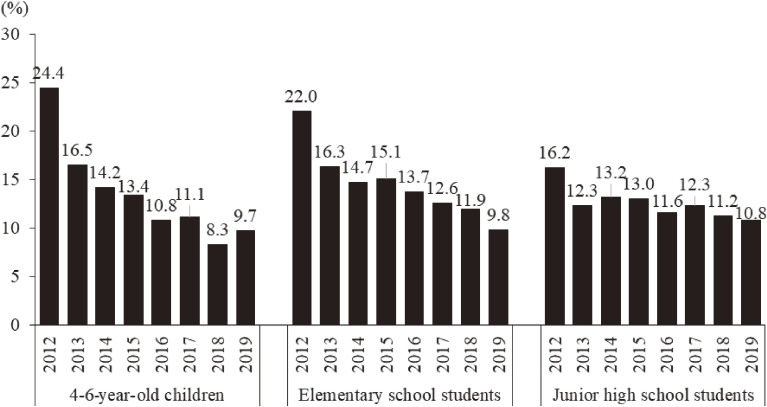
Prevalence of emotional and behavioral problems among children from 2012 to 2019. Children aged 4 to 15 years having some emotional and behavioral problems are identified using the Strength and Difficulties Questionnaire (SDQ) (≥16).

According to the results of the SDQ in three age groups (4–6 years, 7–12 years, and 13–15 years), the prevalence of children with some behavioral and emotional problems was extremely high, being as high as 24.4%, especially in the youngest group (4–6 years) (Figure 8). Though the prevalence decreased rapidly, the prevalence was higher among those staying outside Fukushima than among those staying inside Fukushima in two school-age groups (elementary and junior high school age groups) in 2019, whereas the prevalence among 4–6-year-old children staying inside Fukushima was higher (Figure 3). Meanwhile, one must note that no one in the 4–6-year-old group in 2019 experienced the 2011 disaster.

### Results of interventions

Telephone interventions have been provided for people identified as at high risk based on the results of these core measurements in the survey. This telephone-based support was basically implemented once for a targeted respondent but repeated if requested or necessary. In 2019, 95.0% of the targeted respondents received one-time service, whereas 5.0% received it more than twice. In addition, people were able to phone the team directly if they wished.

The total number of telephone-based interventions from 2012 to 2019 reached 29,956 for adults and 3,334 for children for 7 years. On average, 3,745 interventions for adults and 417 for children were performed every year. In this period (2012–2019), of the above total number of interventions, 55.8% were performed only once (1 year) for a targeted respondent; 21.1% were performed twice (2 years); 10.5% were performed three times (3 years); and 12.6% were performed more than four times (4 years).

The most common complaint expressed during telephone counseling by adults was matters related to physical health, and the next was sleep-related issues. On the other hand, the most common complaint of children was school-related, including refusal to attend school and bullying. In both groups, many respondents receiving the intervention complained of deep concerns over radiation health effects in 2012. They, however, came to have a variety of complaints over time: for example, financial issues, relationships with others, and job-related matters.

## DISCUSSION

### Mental health issues highlighted by the MHLS

The MHLS is a major, long-term, community-based survey that has been expected to contribute to maintaining healthy daily lives of evacuees and making better health policies for them. In order to combine a high-risk approach using telephone-based intervention, the MHLS was launched as an all-inclusive survey, not a randomly sampled one, covering a very large population, more than 210,000, including numerous evacuees staying in various places throughout Japan. In the case of the Chernobyl accident in 1986, which was the same level 7 severity (International Nuclear and Radiological Event Scale) as the Fukushima disaster, no long-term, continuous survey and interventions were implemented for the community population after the accident.^19^ Thus, the MHLS was the first attempt to provide a long-term community-based survey and care after a major nuclear crisis. The main findings and trends obtained from the survey are discussed below, focusing on published articles related to the MHLS.

### Mental health consequences in adults

The prevalence of respondents at high risk of depression and anxiety disorder assessed by the K6 was very high in the first survey year, 2012, compared to that in the general population in Japan.^20^ Furthermore, cross-sectional studies of this first-year survey showed that the K6 was associated with a variety of other variables. For example, the K6 was strongly associated with radiation risk perception about genetic effects,^21^ and this association was also clarified in another longitudinal study analyzing the data obtained during the first 3 years.^22^ Furthermore, the K6 was associated with dietary pattern and appetite loss,^23^^,^^24^ activities of daily living in the aged group,^25^ and bereavement in the young age group.^26^ With regard to longitudinal studies performing trajectory analyses of the K6 in the MHLS, radiation risk perception about genetic effects, sleep satisfaction, and social support predicted deterioration of general mental health status.^22^ K6 was one of the reliable measurements identifying mental health problems, including depression,^9^ often used in different disasters. In the MHLS, the prevalence of respondents at high risk of depression remained high in recent years, whereas they decreased yearly over the first 4 years. Taking into account the large number of disaster-related suicides in Fukushima^27^ and the high standardized suicide mortality ratio,^28^ we should especially focus on affected people at high risk of depression to prevent suicide.

As well as depression, PTSD symptoms are typical psychophysical responses commonly seen in different disasters, which were assessed using PCL and PCL-4 in the MHLS. The prevalence of respondents having probable PTSD reached 21.6% in the first survey year, almost equal to that of rescue personnel after the 9/11 World Trade Center attacks in the United States using the same cutoff value for the PCL.^29^ Whereas the data across the survey years should be compared cautiously because two slightly different types of questionnaires (PCL and PCL-4) were used in the MHLS, the prevalence of probable PTSD was down to nearly 10% in the recent 3 years. Longitudinal studies using PCL data from the MHLS showed that the intensity of PTSD symptoms was associated with being older and difficulties living in evacuation,^30^ and, moreover, PTSD symptoms influenced later radiation risk perception about genetic effects.^31^ Research has reported the possibility of higher probable PTSD prevalence among residents of the Great East Japan Earthquake areas than in the average Japanese population during normal times.^32^ In the Fukushima disaster, many evacuees were thought to be traumatized during the acute phase after the explosions at the Fukushima Daiichi Nuclear Power Plant by experiencing fear and feelings of guilt,^33^ and thus, the posttraumatic responses of the evacuees could be characterized by effects of intense concerns over radiation exposure, as well as their long-term evacuation.

There was also a focus on problem drinking among evacuees mainly using CAGE, because it could increase the risk of suicide or other self-destructive behaviors.^34^ Whereas there are few data regarding the prevalence of problem drinking using CAGE in the general population in Japan, considering significant issues related to suicide in Fukushima as described above,^28^^,^^35^ the latest prevalence of respondents with problem drinking (17.2% in males, 8.2% in females) needs particular consideration. A longitudinal study examining CAGE in the MHLS^36^ showed that risk factors associated with problem drinking depended on sex, economic status in males and history of psychiatric disorders in females. With regard to alcohol consumption in daily life among evacuees based on the MHLS data, a cross-sectional study in the first survey year^37^ clarified the association between general mental health status and change of drinking style before and after the disaster (eg, starting heavy drinking), rather than alcohol consumption itself. According to a longitudinal study during the first 2 years,^38^ predictors of starting drinking after the disaster were being male, 65 years old and less, sleep dissatisfaction, and low general mental health status. A community-based approach for problem drinking based on the above evidence can contribute to reduction of risk of not only psychiatric problems, including suicide, but also alcohol-related physical problems, which led to a high number of disaster-related deaths that reached 2,313 as of September 2021, much higher than in other affected prefectures.^39^

### Radiation risk perception and related issues

In the first survey year, the proportions of respondents having negative risk perceptions about both delayed effects, such as thyroid cancer, and effects across generations, such as genetic ones, were as large as 48.1% and 60.2%, respectively. Both proportions decreased during the first 4 years, but they remained almost unchanged in recent years. In addition, the proportions of those having worries about genetic effects have been larger every year than those having worries about delayed effects. Considering that we have not come to a clear conclusion about the causality between the Fukushima disaster and the onset of thyroid cancer, and that the media has been actively reporting the possibility, it is understandable that there has been a certain number having worries about delayed health effects. We, however, find it difficult to interpret such large proportions of those having worries about genetic effects; generally, the media seems to be very careful to refer to genetic effects from the Fukushima disaster rather than effects on the thyroid.

In either case, particular attention should be paid to the fact that such worries about genetic effects can easily produce self-stigmatization among affected people relating to their pregnancy and/or childcare similar to “Hibakusha” (atomic bomb survivors).^5^ An anti-stigma campaign involving the media, as well as different stakeholders, may mitigate such self-stigmatization among affected people. In addition, studies of the MHLS indicated a strong association between radiation risk perception about genetic effects and different mental health issues including depression,^21^^,^^22^^,^^40^ posttraumatic responses,^31^ and well-being among affected people.^41^ A 3-year longitudinal study of the MHLS^22^ showed that, whereas loss experiences, such as bereavement and house damage, were expected to be severe traumatic events leading to mental health problems in natural disasters, they were not associated with general mental health status; negative radiation risk perception about genetic effects was the most associated. These findings strongly suggest that risk communication and mental health care for affected people should be implemented together in a comprehensive manner. Furthermore, another recent study examining the association between radiation risk perception and relocation showed that radiation risk perception did not dominantly govern whether people were living inside Fukushima Prefecture, but that the locations also affected radiation risk perception.^42^ This suggests that active interaction between people living inside Fukushima and those outside of Fukushima, rather than only risk communication, may encourage evacuees to return to their hometowns.

### Behavioral and emotional effects in children

In the first survey year, the prevalence of children with some behavioral and emotional problems assessed by SDQ was extremely high; for example, the prevalence in the youngest group (4–6 years) was up to 24.4%. This also probably indicates high levels of worry about their children in the mothers, because the SDQ was completed by the parents/guardians living with them, who were the mothers in many cases. Studies performed after the Chernobyl accident showed mental health problems, including depression and PTSD, among mothers due to worries about the radiation health effects on their children as well.^19^ These results suggest that nuclear disasters are likely to elicit intensive, emotional interactions between parents (especially mothers) and their children in affected areas. Thus, further study examining such mother–child interactions after the Fukushima disaster is needed.

According to a cross-sectional analysis in the first survey year, whereas the prevalence of high SDQ children was not associated with the air radiation dose of the area where they experienced the accident,^43^ daily sleep duration was associated with the prevalence of high SDQ children.^44^ Furthermore, a longitudinal study using trajectory analysis during the first 3 years^45^ demonstrated that the prevalence of high SDQ children was strongly associated with peer relationships and daily exercise habit. Related to peer victimization in school such as bullying, the MHLS also showed that nearly 20% of parents (respondents), especially those living with boys, had worries about such victimization of their children.^46^ Taking into account the higher prevalence of SDQ among school-age children in recent years, careful consideration of their needs and school-based care involving teachers are needed.

### Support system linked to the MHLS

Telephone-based interventions have been actively provided for respondents identified as being at high risk of some health problems through the MHLS, and this has been called ‘phone support based on an outreach approach’.^18^ While we have a long history of numerous natural disasters in Japan, the present approach conducted for 210,000 affected people after the Fukushima disaster is a large-scale, long-term one never seen before. It is thought that the telephone-based intervention used in this survey has significant advantages, given that many affected people were dispersed around Japan and forced to live as evacuees for a long time. The telephone-based intervention enables counselors to quickly communicate with many people requiring help regardless of distance when the counselors have enough experience and skills, and collaboration with other care resources is well-established. To evaluate the usefulness of this telephone-based approach, face-to-face interviews with 484 people chosen at random from the target population of the MHLS were performed in 2015.^18^ It was found that the satisfaction rate of those who underwent the telephone-based intervention was up to 74.6%, and, furthermore, such satisfaction with the intervention was significantly associated with positive ideas related to behavioral change.^18^ This telephone-based intervention based on the outreach concept can be feasible and useful in other major disasters affecting a broad area and causing a great number of evacuees, such as the Fukushima disaster. In addition, it can also contribute to enhancing accessibility for affected people, especially those who have difficulty using the internet (eg, elderly people) in the current novel coronavirus disease 2019 pandemic.^47^

### Limitations and future tasks of the MHLS

In the context of the reliability of the survey and the effectiveness of intervention in the MHLS, the biggest issue is low response rates. Whereas the response rates were 40.7% in adults and 63.4% in children in 2012, they remained lower in recent years, at nearly 20%. There are several possible reasons for such low response rates. The first is that, according to the interview survey described above,^18^ the volume of the questionnaires used in the MHLS was too great and possibly imposed a considerable burden on respondents. Given that the MHLS has two roles (one is as a high-risk approach and the other is as an epidemiological study), we may need to perform the survey by separating these roles. For example, a survey for a high-risk approach will be implemented every year with a very limited number of question items useful for interventions, whereas a survey for an epidemiologic study will be done once every few years. This can reduce the burden on respondents, leading to an increase in the response rates. Meanwhile, an interview study^48^ examining differences between respondents and non-respondents in the MHLS showed that the latter group had a significantly higher proportion of psychological distress than the former group. Along with the efforts to increase the response rates described above, consideration should be given to the fact that such response bias can affect the results obtained from the MHLS; that is to say, the annual improvement in K6 scores showed in the present study might not reflect the actual mental health condition among the target population. Second, we may need to focus on a more vulnerable population. For example, considering the high prevalence of people at high risk of psychiatric disorders among those staying outside of Fukushima, focusing on them might be one of the future options. Lastly, compared to the analysis of the results of the MHLS questionnaire, the study of the telephone-based support conducted in the present survey is insufficient. Further analysis of the results of the support, therefore, is also needed in order to understand it better.

We are still facing over 30,000 evacuees, of whom the majority are staying outside Fukushima.^49^ In addition, municipalities having many returnees are experiencing population aging and shortage of care resources.^50^ The MHLS still has an important role, and we need to continue and modify it to meet the current requirements of the affected people and communities.^7^
